# Fertility Limiting Intention and Contraceptive Use among Currently Married Men in Nepal: Evidence from Nepal Demographic and Health Survey 2016

**DOI:** 10.1155/2018/5970705

**Published:** 2018-12-23

**Authors:** Yuba Raj Paudel, Kiran Acharya

**Affiliations:** ^1^Everest College of Nursing, Kathmandu 44600, Nepal; ^2^Karnali College of Health Sciences, Gaushala, Kathmandu 44621, Nepal; ^3^New ERA, Rudramati Marga, Kalopul, Kathmandu 44621, Nepal

## Abstract

**Introduction:**

Less is known about fertility intention of men and family planning (FP) use pattern among men or their spouses who do not want to have more children in Nepal. The objective of the current research was to assess whether number and sex composition of living children determine contraceptive use and method mix among Nepalese men who expressed not wanting to have more children.

**Methods:**

We used couple dataset from NDHS 2016 for the analysis. The sample consisted of 1551 (weighted) men aged 20 or older who had at least one living child and said they wanted no more children. Multinomial logistic regression analysis was conducted to identify determinants of sterilization, traditional, temporary contraceptive use. Analysis was conducted considering clustering and stratification in NDHS 2016 survey.

**Results:**

Of the total respondents, more than 80% mentioned they do not want to have any more children. However, only one-third of the men or their spouses who expressed desire not to have children were using sterilization methods at the time of survey. Contraceptive use showed a strong association with number and sex composition of children with men favouring to have at least 1 or 2 sons. Multinomial logistic regression analysis showed that use of sterilization method (especially female sterilization) was strongly associated with having at least 1 or 2 sons. Men with daughters only and one son with daughters were more likely to use temporary methods.

**Conclusion:**

Among men who do not want to have more children, FP use was associated with number and sex composition of living children. Use of sterilization methods was associated with having at least 1 or 2 sons. Nepal's family planning program can be further strengthened by joining hands with initiatives aimed at promoting the value of girl child.

## 1. Introduction

Despite considerable achievements in the last forty years, Nepal's Family planning (FP) progress has been stalled in the last decade [1–3]. Low use of modern contraceptive has been partly attributed to high spousal separation [4], overreliance on female sterilization and injectables, lower female autonomy, son preference and lower use among rural, marginalized communities, young couples [1, 5–7], and women during postpartum [8] and postabortion period [9].

To date, several studies have investigated barriers of FP use and strategies to improve equitable coverage of FP services in Nepal [6, 10–15]. Elimination of son preference alone was expected to increase contraceptive use by eight percent in Nepal [6]. Some of the previous studies suggested that increasing male involvement could be a game changer in FP program [13, 14, 16]; however, family planning programs operate from the perspective that women are the users and men should support their spouses, often overlooking men's role as users as well as promoters [17].

Like in many traditional societies husbands influence wives' reproductive choice and behavior including use of family planning methods in Nepal [5]. However, husbands' attitude towards contraception use is still not favorable in many communities. Nepal Demographic and Health Survey (NDHS) 2016 showed that more than one in 10 husbands agreed that contraception is women's business and husbands do not need to worry about it [3]. Hence, understanding men's fertility intention and identifying ways for their productive engagement in FP programming and delivery are a current priority.

Previous studies have shown stronger association of son preference with contraceptive use pattern in Nepal compared to other South Asian countries [18, 19]. Furthermore, strong son preference was expressed through increased proportion of permanent method users after two or three sons compared to those having no son [19]. In addition, Leone et al. showed that son preference was expressed through differential stopping of child bearing [6]. Emerging evidence of sex selective abortion and falling sex ratio has been reported after abortion legalization in Nepal [20].

In recent decades, the family planning service landscape has been largely changed in Nepal due to legalization of abortion [21], expansion of public and private health services [22, 23], and improved socioeconomic environment [23]. Although abortion on the ground of sex of the fetus is strictly prohibited, Nepalese couples could have taken advantage of the legalization of abortion to influence sex composition of their children. Furthermore, reduced total fertility rate has been shown to have an impact on son preference [6]. Total fertility rate has been declining in Nepal as shown in the latest rounds of DHS surveys [2, 3]. However, there is little published data from Nepal on contraceptive use pattern according to number and composition of children. Therefore, the current study aimed to investigate fertility intention of men and correlates of FP methods use among men who did not intend to have children. Specifically, we aimed to investigate FP use in relation to number and sex composition of their living children.

## 2. Methods

### 2.1. Data Source

We used data from the 2016 Nepal Demographic and Health Survey (NDHS) [3]. The NDHS 2016 used two-stage stratified cluster sampling to select households. Stratification was done by urban/rural place of residence. In the first stage, Primary Sampling Units (PSUs) were selected using probability proportional to size. In the second stage, households were selected using systematic sampling from individual PSU in rural areas. However, three-stage stratified cluster sampling technique was used for selecting households in urban areas. In the first stage, PSUs were chosen by probability proportional to size followed by random selection of enumeration areas (EA) from PSUs in second stage. In third stage, households were selected systematically from selected EAs. Of total 383 wards selected from all over the country, 184 wards were from urban areas and 199 were from rural areas. A total of 11,473 households (urban, 7294 households, and rural, 4179 households) were selected for the survey. The 2016 NDHS full report was published in 2017 [3].

The 2016 NDHS has a matched couple dataset comprising information on currently married individuals and their spouses. The matched couple dataset was used so that missing information from men's responses can be ascertained from their wives' responses. The dataset comprises 2422 (unweighted) and 2388 (weighted) currently married individuals. Analysis was conducted among weighted sample.

Out of total married men, we excluded men who, at the time of survey, did not have any living children. In addition, we excluded men who were below the age of 20, as well as men who wanted to have more children, or whose wives were pregnant or whose wives had experienced menopause or hysterectomy and whose wives were infecund or subfecund. Thus, our sample included 1551 (weighted) men aged 20 or older who had at least one living child and said that they wanted no more children. The average age at first marriage was 21 years (Standard Deviation: 4.3 years) for men and 17.8 years (Standard Deviation, 3.4 years) for women.

### 2.2. Variables and Their Definition

The outcome variable was contraceptive method used at the time of survey as reported by men. This variable was categorized into five categories: female sterilization, male sterilization, temporary methods (condom, pills, injectables, implant, and IUCD), traditional methods, and nonuse. We used responses from men because men influence the choice of contraceptive methods and we wanted to investigate men's fertility intention. An earlier study showed consistency of husband and wife's responses was high (98%) for permanent methods [15].

We used number of living sons and daughters from women's responses since this information was not available in men's responses. We assumed that the number of living children was from current union. The number and composition of children were categorized as done by Dahal et al. [15]. This categorization reflects the preferred number and sex composition of children in Nepal. Other variables included type of place of residence (urban/rural), provinces (provinces 1 to 7), ecological zones (Mountains, Hills, and Terai), wealth index categories, age categories (20-29, 30-39, 40-49), and ethnicity (Hill Brahamin/Chhettri, Terai Castes, Janajati, Dalit, and others). Similarly, educational status, occupation, decision-making regarding contraception, decision-making on health care, and extent of media exposure were included as covariates.

### 2.3. Statistical Analysis

We conducted descriptive analysis to see background characteristics of men who did not want to have any more children. Further, background characteristics of men who did not want any more children were investigated by current FP methods used. P values from chi-square test were used to see if there were any differences in FP method used by background characteristics. Before fitting the regression model to assess the association of family composition and other background characteristics with use of specific FP methods, we screened all covariates for multicollinearity, and we did not detect collinearity among the variables.

Multinomial logit model was used to identify the determinants of sterilization and temporary and traditional method use. Variables that showed marginal association with current FP use were included in multinomial logistic regression. We further examined if FP use pattern among young cohorts (20-34 years) was different in comparison to overall population. Due to complex sampling design, svyset command was used in STATA version 15.0 accounting for inverse probability weighting, clustering, and stratification to provide unbiased estimates of the population parameters. P values and 95% confidence intervals were presented to indicate statistical association.

## 3. Results

### 3.1. Sample Characteristics

Among surveyed Nepalese men who had at least one living child, more than 8 in 10 wanted no more children. More than half (55%) were aged 20-39 years (Table 1); 62% were from urban areas and 52% were from Terai region of Nepal. Only 17 percent of men had no formal education, whereas 56% had secondary or higher education.

Just over two-thirds (66%) of men who wanted no more children mentioned jointly making decision about family planning with their wives. More than one-third (35%) of the men had had no exposure to family planning messages in the mass media in the last few months of the survey period, while less than 15% had been exposed to messages through radio, TV, or newspapers. The most common family composition among those who did not want more children was one son and one daughter (22%), followed by one or two sons only (19%). The proportion of men who had no living sons was only 5% among those not wanting to have further children. Generally, men who wanted more children tended to have rather small families, or had more daughters than sons.

### 3.2. Contraceptive Use

Among the 1,551 men who wanted no more children nearly a quarter of them (22%) reported not using any contraceptive method at the time of the survey, while 23% reported that their wives had been sterilized. Nine percent had been sterilized themselves, 37% used modern temporary method, and less than 10 percent seem to use traditional method (Table 2). Among 1218 contraceptive users, 47% relied on temporary methods (18% on injectables, 9% on condoms, 10% on pill, 8% on implant, 3% on IUCD) and 12% on traditional methods. Male sterilization constituted 11% of the method mix whereas female sterilization constituted 30% of the mix.

Contraceptive method-mix varied by age-group: Men aged 20-29 reported the lowest levels of male sterilization (1%), while 10% responded that their wives were sterilized and more than half reported to have been using temporary methods (54%). Men aged 40-49 were the most likely to report having used female and male sterilization (32% and 11%, respectively). No use of contraceptive was the highest in those aged 20-29 years (25%). Men with two sons and one daughter were more likely to be using permanent methods than men with other family combinations (54%, three quarters of whom were relying on female sterilization) followed by men who had four children (three sons and one daughter). Male sterilization was common among men who had more than two sons and at least one daughter. The use of sterilization was very low among men who had daughters only. Temporary methods use was the highest among men who had one son and 3 daughters (52%) followed by men who had no living sons (50%). Not using contraception was more common in men who had 1 son and 2 daughters and other category (one son and four daughters, one son and five daughters, and seven or more children).

Contraceptive use also varied by residence especially on female sterilization: A higher proportion of men who lived in rural than in urban areas used female sterilization (27% vs. 21%) while the use of temporary method was the opposite with regard to place of residence, i.e., 39% in urban and 38% in rural. Men living in province 2 (55%) were more likely than other provinces to use female sterilization while men living in province 6 (26%) compared to other provinces were using male sterilization the most. Higher proportion of men in provinces 1 and 4 reported current use of traditional method of family planning compared to those from other provinces. Similarly, men living in the Mountain zone were more likely than those in the Terai to use male sterilization (14% vs.5%) or traditional method (12% vs.8%) and modern temporary method use (49% vs. 29%), while they were much less likely to rely on female sterilization. There were no much differences in no use of contraceptives between Terai and Mountain zone.

Men with higher education had lower use of female sterilizations. Men having secondary education and above (12%) were more likely to report use of traditional method compared to lower education level. The use of contraceptive also differed by occupation: Men working in the professional sector reported the lowest rate of female sterilization (13%) and male sterilization (7%) and highest rate of temporary method use (46%), while no use of contraceptives among men was more common among those involved in agriculture (22%) followed by those involved in clerical/sales/services (21%) and manual skilled/unskilled jobs (20%). Similarly, the use of female sterilization was the lowest in poorest wealth index of household while these men reported the highest use of temporary and traditional methods. Furthermore, there were ethnic differences in contraceptive use among men: Men from Terai castes reported higher use of female sterilization (50%) and reported less use of male sterilization (2%), whereas Hill Bramin/Chettris were the most likely to have been sterilized themselves (15%). Among all ethnic groups, the janajatis were the most likely to use temporary method (44%), and the Muslims/others were most likely to report no use (40%).

The main decision-makers on the use of family planning were found to have an effect on the method choice. The use of temporary methods (52%) was high when the wife was the main decision-maker. Use of male sterilization (23%) and traditional method (23%) were high when husband himself decided on the use of family planning. In addition, when both husband and wife made decision on wife's healthcare then the female sterilizations were more likely to have been used. The use of traditional method was the highest in men when someone else in the household was the decision-maker on spouse's healthcare while use of temporary method was high when the woman herself made decision about her healthcare. Finally, men who had been exposed to family planning messages through all three mass media (radio, TV, and newspaper) were most likely to report use of all family planning methods except female sterilization. No use of contraceptives was the highest among the men who were never exposed to mass media.

Multinomial logistic regression analysis showed that men aged 20-35 years were significantly less likely to use male sterilization methods than no-use compared to older age group (35-49 years) (Table 3). Use of male sterilization was positively associated with having secondary or higher education, being from lower wealth quintiles, and having been exposed to mass media such as radio, TV, and newspaper. It was negatively associated with belonging to provinces 1 and 2 compared to province 7. Male sterilization use was significantly lower when wives made the decisions regarding family planning compared to when husbands made the decision on family planning

According to sex composition of children, men who had sons only, one or two sons only, three or more sons only, and having two sons and one daughter were significantly more likely to have their wives use female sterilization than no use of methods (Table 3). Men with one son and three daughters were significantly more likely to report use of temporary methods than not using any methods in comparison to men from other categories. Overall, these findings indicate a strong propensity to have at least 1 or 2 sons among Nepalese men although they express not wanting to have more children.

Use of traditional method was significantly less when wife made the decision on family planning or when joint decision was made in comparison to when husband made the FP decisions. Use of other temporary methods was lower in provinces 1, 2, 3, and 6 compared to province 7. Further, it was significantly higher among lower wealth quintiles (quintiles 1 and 2) compared to highest wealth quintiles. Hill Brahmin/Chhetri were significantly less likely to use temporary methods than not using any methods.

## 4. Discussion

The aim of the study was to understand fertility intention and family planning use among men who had at least one living child. Although more than 80% expressed no desire to have more children, more than one-fifth of them were not using any FP methods, and 1 in 10 relied on less effective traditional methods. Interestingly, among men who did not want to have any more children, only 5% had daughters only.

Understanding fertility intention and supporting couples to its realization are important from number of perspectives. First, in a context where son preference is high, fertility limiting intention realized by using sterilization methods indicates a degree of consistency between intention and use. Second, women who intended to not have children were more likely to use FP than women who intended to have more children [24]. Third, men/women who did not want to have children but were not using FP methods might represent a group with high level of unmet need for modern contraceptives [15]. Therefore, understanding fertility intention and FP use behavior of couples can be another way to identify populations with higher need and less use of FP than general population.

NDHS 2016 found mean ideal number of children among currently married women to be 2.1 and among currently married men to be 2.2 [3]. However, fertility intention and fertility behavior frequently do not match in Nepal with couples often surpassing their intended family size [25]. A previous study based on NDHS 2001 data found that more than two-thirds of men who had at least 1 child did not want to have more children [15]. Nearly a quarter of these men/or their spouse were not using any FP methods. The authors implied number and sex composition of family favoring son preference, lower level of spousal communication, and service accessibility factors to be main factors for lower use of FP methods (especially male sterilization methods) [15].

In the current analysis, we also found a discrepancy between fertility limiting intention and current use of family planning methods in Nepal. Only one-third of men who reported not intending to have more children were using sterilization methods by themselves or by their wives, 37% were using temporary methods, 9.4% were using traditional methods, and nearly a quarter of them were not using any methods. Using a longitudinal survey data from India, Roy et al. have argued that fertility intentions do not help in predicting fertility behavior [24]. The reasons for inconsistency included desire for more children (33%) mainly due to son preference, having children being up to god (14%), or family/husband influence (13%) to have more children. We believe that the discrepancy between fertility intention and FP use in the current analysis is influenced by deep-rooted son preference, cultural factors, and programmatic factors such as availability of appropriate method mix. Further, we determined fertility intention, and current use of FP from husband's responses, and previous studies have found discrepancy between husbands' and wives' fertility intention and behavior. Covert use or nonuse of FP by women has been documented among Bangladeshi women despite husbands' influence [26]. Therefore, future research on this subject should study both husbands' and wives' fertility preferences.

In addition, the current analysis revealed that son preference affects method-mix because couples do not use sterilization methods until the desired sex composition of their children is attained. Sterilization methods were commonly reported to have been used when there was one or two sons. These results suggest that Nepalese men usually want at least one son and are less likely to resort to sterilization unless they achieve their desired sex composition, even though they report not wanting to have more children. On the other hand, temporary methods were commonly used when they had daughters only or less than two sons. Lower use of sterilization until they have had the desired number of sons was evident among men aged less than 34 years and below indicating that son preference is passed onto younger generations of Nepalese too (data not shown). However, fertility intention is dynamic and depends on many factors such as age, change in marital status, death of children, cultural factors, accessibility of contraceptives, partner influence, and others [27].

Nonuse of family planning methods was more common among couples who had daughters only, and those with one son and two daughters. Although nonuse was less common among men with 1 son and 3 daughters they were the groups most likely to use other temporary methods with lower use of sterilization methods than those having 2 or more sons. This pattern remained similar among men aged below 34 years. A study from Bangladesh showed not having son was significantly associated with nonuse or low use of modern contraceptive methods at parity one or two [28]. At higher parities, they observed an association between lower use of contraceptive among couples who have sons only and couples who had son and daughters. The authors indicated the desire for daughters to be the reason for lower use of contraceptives. However, we did not observe preference for daughter among men with sons only. Men with three or more sons only were more likely to use sterilization methods than couples having less than two sons and at least one daughter. These findings also confirm that son preference is stronger in Nepal than Bangladesh as has been reported previously [18, 19]. Furthermore, the researchers showed an association between use of traditional methods and sex composition of children. In the context of increasing use of traditional methods, future research needs to explore relationship between use of traditional methods and son preference in Nepal.

Furthermore, this analysis showed a regional variation in method mix in Nepal. Use of female sterilization was significantly higher in Terai compared to Mountain and Hill, in province 2 compared to other provinces. Use of male sterilization was least common both in Terai region and in province 2 of Nepal while male sterilization was more prevalent in mountain and Hill. Lower use of female sterilization was compensated by higher use of other temporary methods (mainly female methods) in Mountain and Hill. These findings might reflect programmatic caveats and cultural differences among these regions of Nepal. There is a common belief that vasectomy leads to impurity and is often thought to be equivalent to castration, but it is not clear if this myth is more common in Terai region [29]. Therefore, there is a need to enhance availability of and encourage use of male based FP methods especially in Terai region and province 2 of Nepal. Similarly, availability of female sterilization methods needs to be increased in Mountain and Hill regions along with promotion of male-based methods.

The findings of this study need to be interpreted in light of some limitations. First, we used data from a cross-sectional survey; therefore no temporal relationship could be determined between family composition, background characteristics, and use of specific FP methods. Second, we used responses from men to measure fertility intention and use of FP methods, which could be different from wives' intentions and current use of FP methods. However, we believe that husbands generally influence wives' fertility intention and current use of FP methods in Nepal [30]. Furthermore, there is a 98% consistency between husbands' and wives' responses on use of sterilization methods.

## 5. Conclusion

Son preference was found to be associated with FP use and method mix in Nepal. In light of these findings, family planning service provision in Nepal needs to adopt multidimensional integrated approach. Therefore, in addition to improving the quality and reach of FP services, national family planning program must work in collaboration with efforts to promote the value of girl child. Furthermore, fertility intentions of couples need to be understood during FP services and counseling to support couples to meet their desired number and sex composition. Men need to be involved during FP services and related activities as equal partners of their wives to promote shared decision-making and spousal communication. Longitudinal studies and qualitative research are needed to disentangle spousal dynamics in fertility intention and FP use in relation to number and composition of children.

## Figures and Tables

**Table 1:** Percentage distribution of currently married men aged 20 or older who had at least one living child, by selected characteristics, according to fertility intention, Nepal 2016.

Characteristics	Did not want more children (N=1551)	Wanted more children (N=428)
**Age** **∗** **∗** **∗**		
20-29	10.9	53.4
30-39	43.9	38.4
40-49	45.3	8.2
**Number and sex of living children** **∗** **∗** **∗**		
one or two son's only	18.7	36.4
Two: 1son, 1 daughter	21.6	8.7
Three: 1 son, 2 daughters	11.2	2.0
Three: 2sons, 1 daughter	11.8	1.1
Four:1 son, 3 daughters	4.9	0.0
Four:2 sons,2 daughters	7.5	0.2
Four:3 sons,1 daughter	2.8	0.0
Five:2 sons,3 daughters	3.1	0.3
Five: >=3sons, at least 1 daughter	3.6	0.0
six:>=2 sons, at least 1 daughter	0.8	0.0
Three or more: sons only	3.8	0.3
Daughters only	5.0	50.4
Others	5.0	0.6
**Place of residence**		
Urban	62.4	63.1
Rural	37.6	36.9
**Province**		
Province 1	17.1	20.5
Province 2	20.9	17.9
Province 3	21.7	22.9
Province 4	9.0	8.6
Province 5	16.7	18.7
Province 6	5.5	3.8
Province 7	9.1	7.6
**Ecological zone** **∗**		
Mountain	6.6	3.8
Hill	41.0	46.2
Terai	52.3	49.9
**Education** **∗** **∗**		
Secondary or higher	56.2	73.2
Primary	27.2	19.8
None	16.6	7.0
**Occupation**		
Professional	9.2	12.4
Clerical/sales/services	29.9	27.3
Manual, skilled/unskilled	26.4	30.4
Agriculture	34.4	29.8
**Wealth Index** **∗**		
Poorest	17.8	12.5
Poorer	19.8	18.0
Middle	19.2	18.0
Richer	21.3	28.0
Richest	21.9	23.5
**Ethnicity**		
Hill Brahamin/Chhettri	28.1	28.3
Terai caste	18.4	15.6
Janjatis	38.7	39.4
Dalit	11.3	10.2
Muslim and Others	3.5	6.4
**Decision making on family planning** ^1^		
Mainly wife	19.5	20.6
Wife and husband jointly	66.4	60.8
Self	14.0	18.6
**Decision makers about spouse health care** **∗** **∗** **∗**		
Women Herself	14.5	13.9
Both	43.3	36.6
Self	38.3	32.3
someone else/Others	4.0	17.2
**Exposure to family planning messages in last few months**		
Radio, TV and news paper	14.0	18.5
Any two media	20.3	19.0
One media	30.7	31.5
None	34.9	31.0
Total	100.0	100.0

*∗∗∗*p<0.001, *∗∗*p<0.01, and *∗*p<0.05. Note: p values are based on chi-square test.

^1^Others less than one percent are not shown in the table. For caste, Number of Muslims who did not want more children is 31 and other is 3.

**Table 2:** Percentage distribution of married men who did not want more children, by current contraceptive method, according to selected characteristics.

**Characteristics**	**Female ** **sterilization ** **(N=363)**		**Male ** **sterilization ** **(N=138)**		**Traditional ** **method ** **(N=146)**		**Temporary ** **method ** **(N=571)**		**Nonuse ** **(N=333)**		**Total (N=1,551)**
	%	**95**%** CI**	%	**95**%** CI**	%	**95**%** CI**	%	**95**%** CI**	%	**95**%** CI**	
**All**	**23.4**	**20.5-26.6**	**8.9**	**7.2-11.1**	**9.4**	**7.6-11.6**	**36.8**	**33.4-40.4**	**21.5**	**18.8-24.4**	**100**%
Age*∗∗∗*											
20-29	10.4	6.2-16.9	0.7	0.2-2.2	10.6	6.8-16.2	53.8	45.8-61.6	24.5	17.7-33.0	169
30-39	18.0	14.5-22.0	8.6	5.8-12.7	11.2	8.6-14.4	40.9	36.3-45.7	21.3	17.5-25.7	680
40-49	31.8	27.2-36.8	11.2	8.8-14.1	7.4	5.5-10.0	28.7	23.4-34.6	20.9	17.1-25.3	702
**Number and sex of living children** **∗** **∗** **∗**											
one or two son's only	26.0	19.6-33.5	5.5	3.4-8.9	13.8	8.9-20.6	36.1	28.5-44.4	18.7	13.5-25.3	290
Two:1son, 1 daughter	14.2	10.1-19.4	8.7	5.0-14.7	12.7	9.0-17.6	43.0	36.1-50.2	21.5	16.0-28.2	335
Three: 1 son, 2 daughters	14.3	9.2-21.6	10.6	6.7-16.4	7.8	4.6-13.0	37.3	29.6-45.8	29.9	21.5-39.8	174
Three: 2sons, 1 daughter	39.1	30.6-48.3	14.4	8.4-23.5	5.9	3.5-9.9	24.3	18.3-31.3	16.3	11.3-22.9	183
Four:1 son, 3 daughters	21.4	12.9-33.3	7.4	3.3-15.8	6.3	2.6-14.5	51.6	37.2-65.8	13.2	6.8-24.1	76
Four:2 sons,2 daughters	37.3	27.7-48.0	8.4	4.8-14.5	4.8	2.1-10.6	26.4	19.0-35.3	23.1	15.5-32.9	117
Four:3 sons,1 daughter	38.4	22.4-57.3	13.9	6.8-26.5	2.7	0.7-9.5	32.6	19.4-49.1	12.4	5.2-26.7	43
Five:2 sons,3 daughters	26.9	15.1-43.3	12.3	5.7-24.6	8.9	3.4-20.9	31.0	19.3-45.8	20.9	11.2-35.6	49
Five: >=3sons, at least 1 daughter	18.3	10.1-30.8	12	5.9-22.9	2.5	0.6-9.9	39.9	27.6-53.5	27.4	16.4-41.9	57
six:>=2 sons, at least 1 daughter	34.1	13.1-64.0	17.3	4.8-46.8	7.0	1.0-36.9	15.2	3.6-46.2	26.4	10.2-53.2	13
Three or more: sons only	41.6	28.5-56.0	5.2	2.0-13.2	0.3	0.0-2.5	38.3	25.6-52.9	14.5	7.5-26.3	60
Daughters only	1.7	0.4-7.8	5.0	1.9-12.5	16.4	9.1-27.6	50.3	37.9-62.7	26.6	17.3-38.6	78
Others	17.8	10.4-28.9	6.5	2.8-14.5	10.3	4.9-20.2	34.4	24.4-46.1	31.0	21.3-42.7	78
**Place of residence**											
Urban	21.3	17.7-25.4	9.9	7.4-13.0	9.2	7.0-12.1	39.2	34.6-44.0	20.4	17.2-24.1	969
Rural	27.0	21.6-33.0	7.3	5.1-10.4	9.7	7.0-13.3	32.8	27.7-38.3	23.2	18.8-28.3	583
**Province** **∗** **∗** **∗**											
Province 1	20.0	12.2-30.8	3.4	1.6-7.0	14.2	9.3-21.0	38.9	31.1-47.3	23.5	18.1-29.9	265
Province 2	54.6	47.4-61.7	1.1	0.4-3.3	3.8	1.9-7.2	16.8	12.5-22.3	23.6	18.2-30.0	325
Province 3	8.4	4.7-14.7	15.3	10.2-22.5	8.1	4.2-15.2	43.8	33.4-54.9	24.3	17.1-33.3	337
Province 4	16.2	10.2-24.7	20.4	12.5-31.3	14.3	9.3-21.2	34.9	27.1-43.7	14.2	9.2-21.4	139
Province 5	18.7	13.4-25.5	3.9	2.0-7.4	11.0	7.0-16.8	44.2	36.8-51.9	22.2	15.5-30.7	259
Province 6	7.3	4.5-11.6	26.3	18.5-36.1	7.8	4.5-12.9	40.0	32.5-48.0	18.7	12.1-27.7	86
Province 7	19.5	13.1-28.1	9.3	5.1-16.3	9.8	5.9-16.0	48.1	39.6-56.7	13.2	8.7-19.6	141
**Ecological zone** **∗** **∗** **∗**											
Mountain	2.5	0.8-7.8	13.6	7.3-24.0	11.8	7.8-17.5	48.6	38.2-59.1	23.5	16.5-32.2	103
Hill	10.7	7.9-14.4	13.1	10.2-16.6	11.2	8.0-15.3	45.3	38.9-51.8	19.8	15.5-24.9	637
Terai	36.1	31.1-41.4	5.1	2.9-8.7	7.7	5.7-10.4	28.6	25.1-32.4	22.5	19.0-26.5	812
**Education** **∗** **∗** **∗**											
Secondary or higher	19.4	16.3-22.9	9.8	7.8-12.4	12.1	9.6-15.1	37.8	32.8-43.1	20.9	17.4-25.0	871
Primary	25.4	20.5-31.1	9.5	6.1-14.5	7.0	4.4-10.8	38.3	33.5-43.4	19.8	15.5-24.9	422
None	33.8	26.3-42.2	4.9	2.9-8.2	4.4	2.4-7.8	30.9	25.2-37.2	26.0	20.5-32.4	258
**Occupation**											
Professional	13.1	7.5-21.7	7	3.8-12.6	16	9.6-25.4	46.1	35.3-57.3	17.8	11.2-27.1	139
Clerical/sales/services	23.5	18.7-29.2	10	7.0-14.1	10.6	7.9-14.1	34.4	28.3-41.1	21.4	16.7-27.1	451
Manualskilled/unskilled	27.5	21.9-33.9	8.2	4.4-14.7	7.7	5.1-11.3	36.3	29.7-43.4	20.4	15.7-26.1	398
Agriculture	23.1	19.0-27.8	9.4	6.9-12.8	8.2	5.7-11.6	37.4	32.5-42.5	21.9	18.0-26.5	519
**Wealth index** **∗** **∗** **∗**											
Poorest	9.2	6.0-14.1	12.6	8.9-17.4	10.8	7.0-16.3	46.2	40.8-51.7	21.2	16.5-26.9	276
Poorer	25.2	19.4-31.9	10.7	7.3-15.3	6.8	4.3-10.7	38.8	33.3-44.5	18.6	13.9-24.4	307
Middle	37.5	31.3-44.2	5.4	3.3-8.8	7.4	4.8-11.0	28	22.7-34.1	21.7	16.5-28.0	297
Richer	27.3	21.2-34.3	9.1	4.9-16.3	7.0	4.5-10.8	30.8	24.6-37.8	25.8	20.1-32.5	331
Richest	17.3	11.8-24.7	7.2	4.5-11.4	14.7	10.0-21.2	40.8	31.5-50.9	19.9	14.2-27.1	340
**Ethnicity** **∗** **∗** **∗**											
Hill Brahamin/Chhettri	10.2	6.9-14.9	15.4	11.8-19.8	13.1	10.1-16.7	38.9	33.6-44.5	22.4	18.0-27.5	436
Terai caste	49.6	42.3-57.0	2.1	0.8-5.1	4.7	2.5-8.6	18.0	13.3-23.8	25.7	19.2-33.4	286
Janjatis	19.7	15.1-25.3	8.0	5.1-12.5	10.2	7.4-13.9	43.8	37.5-50.2	18.3	14.5-22.9	600
Dalit	28.2	21.0-36.7	9.8	4.3-20.7	7.0	4.1-11.7	37.6	29.8-46.1	17.4	11.8-24.8	175
Muslim and Others	17.7	8.6-33.0	0		3.7	1.1-12.1	38.7	24.6-55.0	39.9	23.5-58.9	55
**Decision making on Family Planning** ^1^ **∗∗∗**											
Mainly wife	28.7	22.4-35.9	4.9	2.8-8.5	3.3	1.7-6.2	51.6	43.1-59.9	11.5	7.1-18.2	254
Wife and husband jointly	29.0	25.0-33.4	9.6	7.4-12.3	9.2	7.1-11.7	41.2	36.9-45.6	11.0	8.3-14.5	868
Self	14.9	10.1-21.5	23.1	16.1-31.9	22.9	16.3-31.1	29.2	22.3-37.1	10.0	6.0-16.3	182
**Decision makers about spousal health care** **∗** **∗**											
Women herself	12.4	8.0-18.6	10.5	7.1-15.3	14.5	9.8-20.9	40.2	32.5-48.5	22.4	15.7-30.9	224
Both	28.2	23.4-33.5	6.7	4.7-9.4	8.7	6.4-11.6	35.3	31.0-39.7	21.1	17.6-25.2	671
Self	22.1	18.1-26.7	11.4	8.2-15.6	8.5	6.3-11.4	37.3	31.8-43.1	20.7	16.9-25.1	594
Someone else/Others	24.4	15.1-36.9	3.7	1.3-10.0	7.2	2.7-17.7	35.7	23.4-50.1	29.0	18.0-43.1	62
**Exposure to family planning messages in last few months** **∗** **∗** **∗**											
Radio-TV and news paper	10.9	5.8-19.6	15	9.1-23.7	14.6	9.5-21.7	45.6	31.8-60.0	13.9	8.9-21.1	218
Any two media	20.1	15.7-25.3	10.7	6.8-16.3	9.0	6.3-12.9	39.5	33.9-45.3	20.7	15.4-27.3	316
One media	23.5	18.9-28.9	10.3	7.6-13.9	9.0	6.3-12.6	35.2	30.1-40.6	22.0	17.7-27.0	476
None	30.3	25.6-35.5	4.2	2.9-6.2	7.9	5.4-11.4	33.1	28.5-38.0	24.5	20.0-29.5	542

*∗∗∗*p<0.001, *∗∗*p<0.01, and *∗*p<0.05. Note: p values are based on chi-square test.

^1^Others less than one percent are not shown in the table

**Table 3:** Beta coefficients (and standard errors) from multinomial logistic regression analyses assessing associations between selected characteristics and use of specific methods.

**Characteristics**	**Female sterilization vs. no use: beta coefficients(SE)**	**Male sterilization vs. no use: beta coefficients(SE)**	**Traditional vs. no use: beta coefficients(SE)**	**Other temporary method vs. no use: beta coefficients(SE)**
**Age**				
20-35	-1.1(0.32)*∗∗*	-1.20(0.55)*∗∗*	0.07(0.32)	0.25(0.29)
35-49 (ref)	0.00	0.00	0.00	0.00

**No. and sex of living children**				
one or two: sons only	1.80(0.45)*∗∗∗*	-0.51(0.54)	0.91(0.56)	0.51(0.41)
Two: 1son, 1 daughter	0.99(0.52)	-0.45(0.55)	0.69(0.55)	0.35(0.45)
Three:1 son, 2 daughters	-0.02(0.48)	-0.39(0.54)	-0.21(0.56)	-0.13(0.41)
Three:2sons, 1 daughter	1.49(0.47)*∗∗*	-0.01(0.55)	0.13(0.57)	-0.14(0.44)
Four: 1son, 3 daughters	1.99(1.08)	1.59(1.10)	1.65(1.18)	2.66(1.02)*∗*
Four: 2sons, 2 daughters	0.68(0.45)	-0.42(0.62)	-0.39(0.66)	-0.45(0.47)
Three or more sons only	1.94(0.81)*∗*	-0.21(0.95)	-2.19(1.28)	0.96(0.73)
Daughters only	-1.34(0.69)	-0.88(0.72)	0.69(0.67)	0.27(0.51)
Others^1^ (ref)	0.00	0.00	0.00	0.00

**Place of residence**				
Urban	-0.07 (0.34)	0.39(0.38)	-0.36(0.36)	0.33(0.30)
Rural (ref)	0.00	0.00	0.00	0.00

**Province**				
Province 1	-1.60 (0.61)	-2.10(0.71)*∗∗*	-0.84(0.64)	-1.66(0.52)*∗∗*
Province 2	-0.58 (0.63)	-2.85(0.81)*∗∗∗*	-1.4(0.78)	-2.01(0.60)*∗∗*
Province 3	-1.9 (0.65)*∗∗*	-0.13(0.68)	-1.7(0.76)*∗*	-1.80(0.57)*∗∗*
Province 4	-0.51 (0.71)	0.84(0.78)	-0.16(0.77)	-1.01(0.62)
Province 5	-0.43 (0.61)	-0.92(0.74)	-0.02(0.67)	-0.21(0.56)
Province 6	-1.2(0.64)	0.41(0.78)	-1.3(0.73)	-1.29(0.60)*∗*
Province 7 (ref)	0.00	0.00	0.00	0.00

**Ecological zone**				
Mountain	-2.10(0.72)*∗∗*	-0.56(0.70)	0.74(0.65)	0.55(0.49)
Hill	-0.49 (0.40)	-0.28(0.49)	0.61(0.43)	0.57(0.34)
Terai (ref)	0.00	0.00	0.00	0.00
**Education**				
Secondary or higher	-0.18(0.42)	1.01(0.51)*∗*	0.66(0.53)	0.26(0.38)
Primary	0.42(0.45)	0.81(0.53)	0.36(0.49)	0.46(0.41)
None (ref)	0.00	0.00	0.00	0.00
**Occupation**				
Professional	-0.16(0.48)	-0.62(0.56)	0.44(0.51)	0.19(0.44)
Clerical/sales/services	0.58(0.36)	0.39(0.40)	0.33(0.42)	0.04(0.31)
Manual skilled/unskilled	0.48(0.38)	0.56(0.48)	0.63(0.46)	0.44(0.35)
Agriculture (ref)	0.00	0.00	0.00	0.00
**Wealth quintile**				
Lowest	0.74(0.58)	2.4(0.68)*∗∗∗*	1.15(0.63)	1.37(0.50)*∗*
Second	1.8(0.51)*∗∗*	2.6(0.63)*∗∗∗*	0.73(0.55)	1.52(0.42)*∗∗∗*
Middle	0.91(0.43)	1.13(0.60)	0.07(0.49)	0.47(0.37)
Fourth	0.25(0.33)	1.2(0.55)*∗*	-0.41(0.41)	0.29(0.30)
Highest	0.00	0.00	0.00	0.00
**Ethnicity**				
Hill Brahamin/Chettri	-1.2(0.49)	-0.36(0.57)	-0.46(0.49)	-0.93(0.46)*∗*
Janajatis	-2.5(0.32)*∗*	-0.30(0.53)	-0.08(0.43)	-0.15(0.37)
Other	0.00	0.00	0.00	0.00
**Decision making on family planning**				
Mainly wife	0.66(0.49)	-1.77(0.54)*∗∗*	-2.3(0.59)*∗∗∗*	0.60(0.46)
Wife and husband jointly	0.49(0.43)	-0.72(0.41)	-0.83(0.39)*∗*	0.58(0.39)
Self	0.00	0.00	0.00	0.00

**Decision makers about spousal health care**				
Women herself	-0.59(0.43)	-0.12(0.45)	0.46(0.45)	-0.12(0.38)
Both	-0.14(0.32)	-0.30(0.37)	-0.36(0.35)	-0.09(0.30)
Someone else/others	-0.31(0.62)	-0.47(0.78)	-0.27(0.74)	-0.19(0.58)
Self	0.00	0.00	0.00	0.00

**Exposure to family planning messages in last few months**				
Radio, TV and news paper	0.22(0.55)	1.68(0.60)*∗∗*	0.75(0.54)	0.90(0.47)
Any two media	-0.45(0.34)	0.56(0.40)	-0.25(0.44)	0.08(0.30)
One media	0.07(0.31)	0.72(0.42)	0.75(0.54)	0.07(0.29)
None	0.00	0.00	0.00	0.00
Intercept -2 log-likelihood	0.69(0.91)	-0.85(1.1)	0.33(1.07)	0.58(0.86)

*∗*p<0.05, *∗∗*p<0.01, and *∗∗∗*p<0.001. ^1^Including families with three sons and one daughter, with five or six children of both sexes, and with seven children.

Note: ref=reference; SE =standard error.

## Data Availability

Since this is a further analysis of 2016 Nepal Demographic and Health Survey, data used in this analysis can be obtained upon request from DHS website https://dhsprogram.com/data/.
